# Dynamic musical communication of core affect

**DOI:** 10.3389/fpsyg.2014.00072

**Published:** 2014-03-17

**Authors:** Nicole K. Flaig, Edward W. Large

**Affiliations:** Music Dynamics Lab, Department of Psychology, University of ConnecticutStorrs, CT, USA

**Keywords:** neurodynamics, consciousness, affect, emotion, musical expectancy, oscillation, synchrony

## Abstract

Is there something special about the way music communicates feelings? Theorists since Meyer (1956) have attempted to explain how music could stimulate varied and subtle affective experiences by violating learned expectancies, or by mimicking other forms of social interaction. Our proposal is that music speaks to the brain in its own language; it need not imitate any other form of communication. We review recent theoretical and empirical literature, which suggests that all conscious processes consist of dynamic neural events, produced by spatially dispersed processes in the physical brain. Intentional thought and affective experience arise as dynamical aspects of neural events taking place in multiple brain areas simultaneously. At any given moment, this content comprises a unified “scene” that is integrated into a dynamic core through synchrony of neuronal oscillations. We propose that (1) neurodynamic synchrony with musical stimuli gives rise to musical qualia including tonal and temporal expectancies, and that (2) music-synchronous responses couple into core neurodynamics, enabling music to directly modulate core affect. Expressive music performance, for example, may recruit rhythm-synchronous neural responses to support affective communication. We suggest that the dynamic relationship between musical expression and the experience of affect presents a unique opportunity for the study of emotional experience. This may help elucidate the neural mechanisms underlying arousal and valence, and offer a new approach to exploring the complex dynamics of the how and why of emotional experience.

## INTRODUCTION

Every known civilization creates unique and sophisticated musical forms to communicate affect, and humans consciously seek out musical experiences because of the feelings they evoke. There is widespread agreement that music induces emotional experiences, and that at least some aspects of this phenomenon are universal. But what is the nature of musical feelings and what is the relationship between musical feelings and emotions? Over the years, competing theories have been developed to address these issues, and sophisticated experimental paradigms have been devised to investigate them. However, incommensurate claims and variable findings leave many open questions. Are affective responses to music accidents of evolution? Is there something special about musical communication? Can the study of emotion teach us anything about the nature of music? And what, if anything, can music teach us about the nature of emotional experience?

Since Meyer’s (1956) pioneering work in music and emotion, theorists have struggled to explain how music could stimulate emotional experiences by somehow triggering basic, evolutionarily ancient psychological processes tied to survival. Empirical approaches have sought specific behavioral and/or physiological responses to music (Juslin and Sloboda, 2001). One specific problem that arises is the question of how an object-directed emotion can be evoked by music with no external referent. Another is how a sophisticated, non-referential form of cultural expression, learned over many years of exposure, could engender innate, survival-related responses.

The goal of this paper is to stake out some new territory in the debate on musical emotion. First, we will ask whether emotional experiences are really “basic” (Allport, 1924; Izard, 1971), or whether they are psychologically constructed from domain-general processes (Barrett, 2009a). Next, we will explore the idea that all conscious systems consist of multiple neural processes, produced by spatially dispersed events in the physical brain and integrated into a seamless neurodynamic whole through synchrony of neural oscillations (Edelman, 2003). Core affect is thought to be one primitive, domain-general aspect of the dynamic core of consciousness (Russell, 2003) especially relevant to emotional experience (Barrett, 2009a). Then, we will review recent evidence that neuronal synchrony with music gives rise to musical qualia including tonal and temporal expectancies (Large and Jones, 1999; Lerdahl, 2001; Huron, 2005; Large, 2010a). Finally, we will argue that music-synchronous responses couple into the dynamic core of consciousness, directly modulating core affect.

## THEORIES OF MUSIC AND EMOTION

Music does not have obvious survival value (cf. Pinker, 1997) and yet is able to elicit strong emotional reactions. Many biological perspectives consider the primary function of emotion as a response to behavioral demands that may require mobilization for action; they evolved to prepare an individual to deal with situations that were significant for survival and reproduction (Darwin, 1872/1965; Cosmides and Tooby, 2000; Huron, 2005, 2006). Darwin (1872/1965) proposed that the origin of the musical communication of emotion was to be found in the evolutionary process of sexual selection.

Psychological approaches to musical emotions have been heavily influenced by the theory of basic emotion. Basic emotion theorists have sought to identify categories of emotions that share distinct collections of properties such as patterns of autonomic nervous system activity and behavioral responses or action tendencies (Allport, 1924; Izard, 1971; Ekman, 1972; Panksepp, 1998). Other approaches consider emotions to be constructed from more primitive processes, including affect (Irons, 1987; Ortony et al., 1988; Russell, 2003; Barrett, 2006, 2009b; Duncan and Barrett, 2007). Mechanisms of musically induced emotion have been explored at great length with varying causations, interpretations, and results.

Meyer’s (1956) approach to musical emotion has been highly influential in part because he was the first to seriously take into account both philosophical works (e.g., Langer, 1951) and psychological theories of emotion (cf. Dewey, 1895; MacCurdy, 1925; Angier, 1927; Rapaport, 1950). Meyer observed that namable emotions are – unlike music – event-directed, and that emotional experiences are much more subtle than the “crude and standardized words we use to denote them.” He also observed that emotional responses are not innate, they are highly variable, and they depend on learning and enculturation (Meyer, 1956). He therefore concluded that, for the most part, music does not communicate genuine emotions. “That which we wish to consider” wrote Meyer, “is that which is most vital and essential in emotional experience, the feeling-tone accompanying emotional experience, that is, the affect” (Meyer, 1956, p.12).

Meyer was also the first to suggest that what is now called statistical learning applies to music and determines musical feelings. For example, a passage of tonal music leads to the feeling that some pitches are more stable than others. More stable pitches are felt as points of repose, and less stable pitches are felt to point toward, or be attracted to the more stable ones (Lerdahl, 2001). Such relationships are reflected in naming conventions of many musical cultures, including Western, Indian and Chinese (Meyer, 1956). Based on the failure of earlier attempts to account for musical communication based on vibrations, ratios of intervals, and so on, he argued that feelings of stability and attraction are learned through experience with the music of a particular culture. Moreover, the associations of musical moods, such as happy and sad, with major or minor harmonies, or the affective qualities associated with ragas in North Indian tonal systems are conventional designations, having little to do with the sound itself (Meyer, 1956).

Finally, Meyer (1956) argued that the frustration of expectancy is the basis for affective responses to music. He believed that affect is aroused when an action tendency is inhibited. Music, unlike other emotional stimuli, is not referential; it both creates and inhibits expectancies thereby providing meaningful and relevant resolutions within itself. Music communicates affect through violations and resolutions of learned expectancies.

The latter two points were taken up by modern empiricists and theorists, who studied musical expectancy and statistical learning in a variety of musical domains, and from various points of view (e.g., Krumhansl, 1990; Narmour, 1990; Large and Jones, 1999; Tillmann et al., 2000; Lerdahl, 2001; Huron, 2005; Temperley, 2007). Perhaps the most comprehensive attempt to extend Meyer’s expectancy theory of musical emotion is Huron’s “Sweet Anticipation” (2006). Huron argues that a fundamental job of the brain is to make predictions about the world, and successful predictions are rewarded. Within tonal context, the most stable pitches are experienced as most pleasant; within a metrical context, events that occur at expected times are more pleasurable. Thus, Huron argues, music is fundamentally a hedonic experience.

Huron emphasizes that tonal and temporal expectancies in music are learned. Musical events evoke distinctive musical qualia, and Huron reviews the body of empirical evidence showing that qualia such as stability and attraction (“scale degree qualia”) correlate with statistical properties of music (Huron, 2006). He agrees with Meyer that the associations of major and minor modes with happy and sad qualia are learned associations.

In a key break from Meyer, however, Huron argues that expectancy evokes emotions, not merely affect. He adopts a two-process approach, which posits a fast time-scale *reaction* and a slow time-scale *appraisal* (LeDoux, 1996). Specific emotional responses involve primitive circuits that are conserved throughout mammalian evolution, and function relatively independently of cognitive circuits (LeDoux, 2000). He hypothesizes, for example, that unexpected events in music activate the neural circuitry for fear, leading to the feeling of surprise. He goes so far as to suggest that basic survival-related responses, including fight, flight and freezing, lead to the specific subjective musical experiences of frisson, laughter, and awe, respectively.

Juslin and Vastfjall (2008), consider emotions to be affective responses that involve subjective feelings, physiological arousal, expressions, and action tendencies. They too, reject Meyer’s claim that music does not induce genuine emotions, because musical responses can display all these features. They endorse the notion that emotions involve intentionality^1^; emotions are “about” something. However, they claim that music induces a wide range of both basic and complex emotions because music triggers a variety of psychological mechanisms beyond expectancy. They go on to describe how brain stem reflexes, evaluative conditioning, visual imagery, episodic memory, and emotional contagion can lead to genuine emotional responses. Brain stem reflexes trigger emotional responses because acoustic characteristics are taken by the brain stem to signal an urgent event. Evaluative conditioning is a special kind of classic conditioning in which a stimulus without an emotional meaning, e.g., music, is consistently paired with an emotional experience, eventually coming to trigger the emotional response. Presumably, the learned pairing of major with happy and minor with sad (Meyer, 1956; Huron, 2006) would be an example of this phenomenon. Triggering of visual imagery as well as episodic memories (Janata et al., 2007) can also lead to emotional experiences (Sloboda and O’Neill, 2001). In all of these cases the emotional responses are intentional – they are about something.

Emotional contagion, in Juslin and Vastfjall’s (2008) view, is a process in which a listener perceives the emotional expression of the music, and then “mimics” this expression internally, as with other forms of interpersonal interaction like bodily gestures, facial gestures, and speech (Juslin and Vastfjall, 2008). In their conceptualization, music evokes basic emotions with distinct nonverbal expressions (Juslin and Laukka, 2003; Laird and Strout, 2007), and this process operates similarly to emotional contagion via facial and vocal expressions of emotion (Tomkins, 1962; Ekman, 1993). Emotional contagion is linked to activation of the so-called mirror neuron system (Rizzolatti and Craighero, 2004), and Juslin (2001) suggests that music can operate in this way because in some sense it imitates other forms of social interaction. Below we will offer a somewhat different view of contagion.

If we consider the full spectrum of phenomena discussed by Juslin and Vastfjall (2008), it seems clear that a wide range of emotional responses can be triggered by music. However, from a musical point of view, some of these mechanisms are more interesting than others. Loud unexpected sounds can frighten us, and auditory stimuli can trigger conditioned responses. But in these examples, music serves merely as a trigger. Many other kinds of stimuli can trigger such responses equally well; they need not be musical or even auditory (e.g., LeDoux, 2000). Episodic memory and visual imagery likely account for a significant proportion of the emotional responses people experience on a day-to-day basis (Janata et al., 2007). Moreover, there are a many reasons to believe that music is especially effective at eliciting episodic memories (Janata et al., 2007; Eschrich et al., 2008). If we could understand the fundamental mechanisms of musical communication, this may help us to understand why episodic memories are so effectively evoked by music. Here, we take a different approach to understanding the ability of music to elicit feelings, one that does not treat music merely as a trigger but rather focuses on fundamental dynamic mechanisms of affective communication.

The remainder of this article is concerned primarily with musical expectancy and contagion, as these are mechanisms that seem to us to be most inherently musical. Expectancies arise in response to complex, explicitly musical structures such as tonality and meter. Contagion is a kind of empathic resonance (Molnar-Szakacs and Overy, 2006; Chapin et al., 2010) that enables music to function as a type of interpersonal communication. Our approach will be to link expectancy and contagion with the dynamics of the physical brain. This involves addressing several basic questions. What is the nature of emotional experience: are emotions basic, evolutionarily adapted and cross-cultural, or are emotions constructed from more fundamental psychological ingredients? Are musical qualia based solely on learned contingencies, or do they arise from intrinsic neurodynamics? And what is the nature of the relationship, such that music is able to elicit affective experiences?

## EMOTION, AFFECT, AND CONSCIOUSNESS

Influenced by Darwin’s (1872/1965) theory of pan cultural emotions, Tomkins (1963) and Ekman et al. (1987) argued that emotions are genetically determined products of evolution. Basic emotions are discrete, and each category shares a distinctive collection of properties including patterns of autonomic nervous system activity, behavioral responses or action tendencies, and a set of emotion-specific brain structures that are thought to mediate these particular “basic” emotions. Each basic emotion derives from a particular causal mechanism; an evolutionarily preserved module in the brain (Tomkins, 1963; Ekman, 1992; Panksepp, 1998). Ekman (1984) proposed that the natural boundaries between types of emotion could be determined by differences in facial expression. Huron’s (2005) approach to musical emotion and Juslin and Vastfjall’s (2008) multiple mechanisms theory tend to endorse the basic emotion view. Basic emotions are cross-cultural and non-basic emotions are specific to cultural upbringing. However, there is little agreement about which emotions are basic, how many emotions are basic, and how basic emotions are defined.

Recent behavioral, psychophysiological, and neural findings (e.g., Barrett, 2006; Pessoa, 2008; Lindquist et al., 2012) have led a number of emotion theorists to question the basic emotion view (Ortony and Turner, 1990; Russell and Barrett, 1999; Duncan and Barrett, 2007). An alternative approach holds that diverse human emotions result from the interplay of more fundamental domain-general processes (Russell, 2003; Pessoa, 2008; Barrett, 2009b). Psychological constructionists argue that emotions are culturally relative, learned, and, though they are a result of evolution, they are not biologically basic (Russell and Barrett, 1999; Duncan and Barrett, 2007). Emotions are the combination of psychologically primitive processes that encompass both affective and intentional components. A specific emotion is not the invariable result of activation in a particular brain area; neural circuitry realizes more basic processes across emotion categories (Pessoa, 2008; Wilson-Mendenhall et al., 2013). Meyer’s approach to the musical communication of affect is consistent with this view.

Contemporary neurodynamic approaches hypothesize that all conscious states are a multimodal process entailed by physical events occurring in the brain (Tononi and Edelman, 1998; Engel and Singer, 2001; Searle, 2001; Seth et al., 2006; Pessoa, 2008). The neural structures and mechanisms underlying consciousness contribute domain-general processes to many psychological phenomena. Importantly, when spatially distinct areas contribute to the contents of consciousness, they enter into a unified neurodynamic core (e.g., Edelman, 2003). Neurodynamic theories of consciousness propose that the synchronous activations of the thalamocortical system give rise to the unity of conscious experience (Edelman and Tononi, 2000; Varela et al., 2001; Cosmelli et al., 2006). Binding of spatially distinct processes is thought to occur through enhanced synchrony in gamma and beta band rhythms (Engel and Singer, 2001; Fujioka et al., 2012), and high frequency activity is modulated by slower rhythms such as delta and theta (Lakatos et al., 2005; Buzsáki, 2006; Canolty et al., 2006).

Intentionality and affect are fundamental properties of conscious experience (Searle, 2001). Conscious processes point to or are about something (Brentano, 1973; Searle, 2000), and they possess a valence and a level of activation (Barrett, 1998, 2006; Searle, 2000). Searle’s theory of consciousness (Searle, 1992, 2004), Edelman’s dynamic core theory (Edelman, 1987; Edelman and Tononi, 2000) and Damasio’s somatic marker hypothesis (cf. Damasio, 1999) all emphasize dynamic processes that encompass both intentionality (e.g., appraisal, see Scherer, 2001; Smith and Kirby, 2001) and affect (Russell and Barrett, 1999; Davidson, 2000). An emotional experience includes affect as one important ingredient, but intentional psychological processes – perception, cognition, attention, and behavior – are also necessary (Pessoa, 2008; Barrett, 2009b). To a great extent, the difference between an emotion and a cognition depends on the level of attention paid to the core affect (Russell, 2003).

Affect can be characterized as fluctuating level of valence (pleasure/displeasure) and arousal (activation/deactivation; Wundt, 1897; Russell, 2003; Barrett and Bliss-Moreau, 2009). It is the most elementary consciously accessible sensation evident in moods and emotions**(Russell, 2003). Core affect is so called because it is thought to arise in the core of the body or in neural representations of body state change (Russell and Barrett, 1999; Russell, 2003). It has been observed in subjective reports (Barrett, 2004), in peripheral nervous system activation (Cacioppo et al., 2000), and in facial and vocal expression (Cacioppo et al., 2000; Russell, 2003). The experience of core affect is thought to be present in infants (Lewis, 2000) and psychologically universal (Russell, 1991; Mesquita, 2003).

**FIGURE 1:**
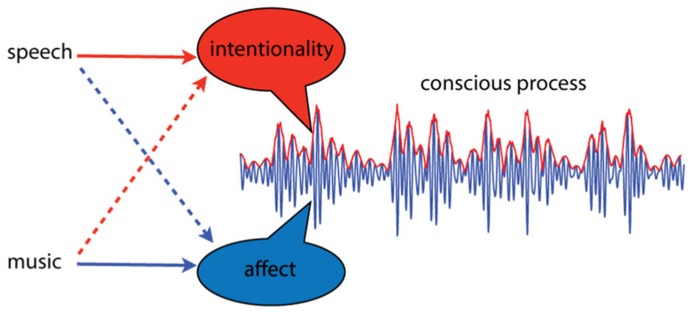
**A conscious process is conceived as a dynamic pattern of activity.** Intentionality and affect are conceived as separable aspects of such processes, and different types of communication sounds may convey more of one or the other. Language primarily communicates intentionality; it is “about” events in the external world. However, certain aspects of speech, such as prosody, can communicate affect. Music, on the other hand, communicates primarily affect; it is most often not “about” anything. However, under certain circumstances music can signify events or elicit memories.

Intentional thought and affective experience are thought to arise as dynamic aspects of spatially distinct dynamic processes, integrated through synchrony of neural oscillations (Tononi and Edelman, 1998; Searle, 2001; Seth et al., 2006). Let us attempt to illustrate this idea, emphasizing dynamic over spatial aspects, by integrating over neural location. The result is an average, summarizing the activity of multiple brain areas, as shown in **Figure 1**. The dynamic properties of this pattern are the critical features; intentionality and affect correspond to dynamic aspects of the integrated neural activity. In this illustration, affective aspects correspond to changes in higher frequency activity, while intentional aspects take place at lower frequencies, and appear as amplitude modulations. This is only a visual aid of course; we do not know enough to speculate about which frequency bands or dynamic features might correspond to intentionality and affect. Here we oversimplify to illustrate the point that relevant aspects of experience may correspond to dynamical aspects of integrated neural processes. If this approach is on the right track, however, then this way of thinking about core affect may lead to a better understanding why music is such an especially effective means of affective communication.

## MUSICAL NEURODYNAMICS

At any given moment, a unified neurodynamic process is shaped by exogenous sensory input such as sights or sounds, input from the body such as vestibular sensations, endogenous constructs such as autobiographical memories, and by communication sounds such as music and speech. It seems likely that if intentionality and affect are different dynamic aspects of these spatiotemporal patterns, then different kinds of communication sounds may couple into different aspects of the dynamics. Of course, it is well established that different modes of auditory communication, i.e., music and speech, convey more of one aspect or the other. Speech primarily communicates intentionality; it is “about” events in the external world. Nevertheless, certain aspects of speech, such as prosody, directly communicate affect. Music, on the other hand, communicates primarily affect; it is most often not “about” anything. However music can signify objects or events, and it can evoke memories and images. Thus, both types of signals can induce emotions, although in different ways. What we want to suggest is that music may couple directly into affective dynamics because it causes the brain to resonate in certain ways.

**FIGURE 2:**
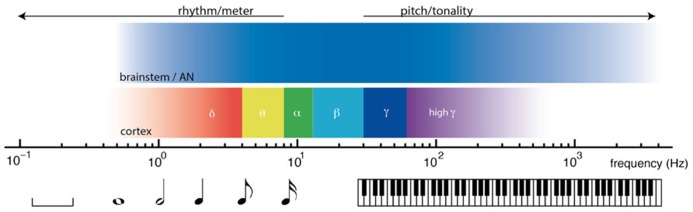
**Timescales of neural dynamics and timescales of musical communication.** Music engages the brain on multiple timescales, from fractions of one cycle per second (left) to thousands of cycles per second (right). At rhythmic timescales, cortical oscillations entrain to rhythmic stimuli. At tonal timescales, volleys of action potentials time lock to acoustic waves.

Nonlinear oscillation and resonant responses to acoustic signals are found at multiple time scales in the nervous system, from thousands of Hertz in the auditory nerve and brainstem, to cortical oscillations in delta, theta, beta, and gamma ranges. The relative timescales of these processes are illustrated in **Figure 2**. From the earliest stages of the auditory system, volleys of action potentials time-lock to dynamic features of acoustic waves (Joris et al., 2004; Laudanski et al., 2010). Time-locked brainstem responses are thought to be important in the perception of pitch, which is observed from 30 Hz (Pressnitzer et al., 2001) up to about 4000 Hz (Plack and Oxenham, 2005). In auditory cortex, endogenous cortical oscillations entrain to low frequency rhythms of acoustic stimuli (Lakatos et al., 2008; Nozaradan et al., 2011). Cortical entrainment is thought to be important in the perception of rhythm, which extends from about 8 Hz (Repp, 2005a) to ultra low cortical frequencies (Buzsáki, 2006; Large, 2008). Between the timescales of pitch and rhythm lie the frequencies thought to be important in binding neural processes into unified conscious scenes (Engel and Singer, 2001; Seth et al., 2006; Fujioka et al., 2012).

### PITCH AND TONALITY

In central auditory circuits, action potentials phase- and mode-lock to the fine time structure and the temporal envelope modulations of auditory stimuli at many different neural levels (Langner, 1992; Large et al., 1998; Joris et al., 2004; Laudanski et al., 2010). Neural synchrony is thought to be important in pitch perception (Cariani and Delgutte, 1996; Hartmann, 1996), consonance (Ebeling, 2008; Shapira Lots and Stone, 2008), and musical tonality (Tramo et al., 2001; Large, 2010a). While phase-locking is well established, mode-locked spiking patterns have recently been reported in the mammalian auditory system (Laudanski et al., 2010) and may explain the highly nonlinear responses to musical intervals that can be measured in the human auditory brainstem response (Lee et al., 2009; Large and Almonte, 2012; Lerud et al., 2013).

Mode-locking implies binding between neural frequencies that display particular frequency relationships (Hoppensteadt and Izhikevich, 1997). In this form of synchrony a periodic stimulus interacts with intrinsic neural dynamics causing *m* cycles of the oscillation to lock to *k* cycles of the stimulus. Mode-locking leads to neural resonance at harmonics (k^*^f1), subharmonics (*f1*/*m*), summation frequencies (e.g., *f1* + *f2*), difference frequencies (e.g., *f2 *- *f1*), and integer ratios (e.g., k^*^f1/*m*)^2^. This implies feature binding based on harmonicity (Bregman, 1990), and suggests a role for mode-locking in the perception of pitch (cf. Cartwright et al., 1999). This also predicts a significant cross-cultural musical invariant (Burns, 1999) because octave frequency relationships (2:1 and 1:2) are the most stable, followed by fifths (3:2), and fourths (4:3). Mode-locking may provide a neurodynamic explanation for musical consonance and dissonance (Shapira Lots and Stone, 2008) that does not depend on interference (e.g., Plomp and Levelt, 1965).

Perhaps most relevant to the current discussion is the issue of scale degree qualia (Huron, 2006), which has important implications for understanding musical expectancy (Meyer, 1956; Zuckerkandl, 1956). Scale degree qualia differentiate musical sound sequences from arbitrary sound sequences, and are thought to enable non-referential sound patterns to carry meaning. Most discussions of expectancy and emotion assume scale degree qualia to be learned based on the statistics of tonal sequences (e.g., Meyer, 1956; Krumhansl and Kessler, 1982; Lerdahl, 2001; Huron, 2006), and therefore culture-dependent. However, recent dynamical analyses have shown that mode-locking provides a better explanation for quantitative measurements of stability in both Western and North Indian tonal systems (Krumhansl and Kessler, 1982; Castellano et al., 1984; Large, 2010a; Large and Almonte, 2011). Thus, scale degree qualia likely depend on the interaction of the stimulus sequence with intrinsic neurodynamic properties of the physical brain.

### RHYTHM AND METER

At the timescale of rhythm and meter, relationships between musical and neural rhythms are equally striking (Musacchia et al., 2013). In auditory cortex, brain rhythms nest hierarchically, for example delta phase modulates theta amplitude, and theta phase modulates gamma amplitude (Lakatos et al., 2005). Like neural rhythms, music rhythms nest hierarchically, such that faster metrical frequencies subdivide the pulse (London, 2004). Pulse perception provides a good match for the delta band (0.5–4 Hz, see London, 2004) while fast metrical frequencies occupy theta (4–8 Hz, see e.g., Repp, 2005b; Large, 2008). Importantly, acoustic stimulation in the pulse range synchronizes auditory cortical rhythms in the delta-band (Will and Berg, 2007; Stefanics et al., 2010; Nozaradan et al., 2011) and modulates the amplitude of higher frequency beta and gamma rhythms (Snyder and Large, 2005; Iversen et al., 2006; Fujioka et al., 2012). Models of synchronization to acoustic rhythms (see e.g., Large, 2008) have successfully predicted a wide range of behavioral observations in time perception (Jones, 1990; McAuley, 1995), meter perception (Large and Kolen, 1994; Large, 2000), attention allocation (Large and Jones, 1999; Stefanics et al., 2010), and motor coordination (Kelso et al., 1990; Repp, 2005c). Moreover, musical qualia including metrical expectancy (Huron, 2005), syncopation (London, 2004), and groove (Tomic and Janata, 2008; Janata et al., 2012), have all been linked to synchronization of cortical rhythms and/or bodily movements. In addition, synchronization of rhythmic movements to music (Burger et al., 2013) and synchronization between individuals (e.g., Hove and Risen, 2009) have been linked to affective responses.

The perception of rhythm also provides an example of synchronous time-locked patterns of activity integrating the function of multiple brain regions. When people listen to musical rhythms that have a pulse or basic beat, multiple brain regions are activated, including auditory cortices, cerebellum, basal ganglia, premotor cortex, and the supplementary motor cortex (Zatorre et al., 2007; Chen et al., 2008; Grahn and Rowe, 2009). In these areas, the amplitude of beta band activity waxes and wanes with the pulse of the acoustic stimulation (Snyder and Large, 2005; Iversen et al., 2006; Fujioka et al., 2012). The specific neural structures involved depend on the tempo of the stimulus, and it appears that the synchrony of beta band processes is what binds the neural activity (Fujioka et al., 2012). This suggests that perhaps it is not the areas *per se*, but the integrated neural activity that corresponds to the experience of pulse.

## MUSICAL COMMUNICATION AS NEURODYNAMIC RESONANCE

We can summarize the above discussion by saying that music taps into brain dynamics at the right time scales to cause both brain and body to resonate to the patterns. This causes the formation of spatiotemporal patterns of activity on multiple temporal and spatial scales within the nervous system. The dynamical characteristics of such spatiotemporal patterns – oscillations, bifurcations, stability, attraction, and responses to perturbations – predict perceptual, attentional, and behavioral responses to music, as well as musical qualia including tonal and rhythmic expectations. Conceptualization of consciousness in similar neurodynamic terms leads to a new way to think about how music may communicate affective content. Neurodynamic responses that give rise to musical qualia also resonate with affective circuits, enabling music to directly engage the sorts of feelings that are associated with emotional experiences. In this section we ask, how might affective resonance take place, do musical qualia arise from intrinsic neurodynamics, and what exactly is communicated?

### AFFECTIVE RESONANCE

We begin with an example of affective resonance to rhythm. Expressive piano performance is a kind of social interaction in which correlated fluctuations in timing and intensity transfer emotional information from the performer to the listener (Bhatara et al., 2010). Expressive tempo fluctuations display *1/f *structure (Rankin et al., 2009; Hennig et al., 2011), and listeners predict such tempo changes when entraining to musical performances (Rankin et al., 2009; Rankin, 2010). A recent study compared BOLD responses to an *expressive* performance and a *mechanical* performance, in which the piece was “performed” by computer, with no fluctuations in timing and intensity. Greater activations were found in emotion and reward related areas for the expressive performance, consistent with transfer of affective information. Tempo fluctuations, BOLD activations and real-time ratings of valence and arousal were also compared for the expressive performance. Over the 3–1/2 min performance, fluctuations in timing correlated with BOLD changes in motor networks known to be involved in rhythmic entrainment, and in a network consistent with the human “mirror neuron” system (Chapin et al., 2010). As tempo increased, activation in these regions increased. Tempo fluctuations also correlated with real-time reports of affective arousal.

Despite the fact that the tempo-correlated activations were observed in so-called mirror neuron areas, this was not motor mirroring; half the participants were not musicians, and none were familiar with the piece. Could listener responses arise from a more general form of contagion in which the perception of affective expression directly induces the same emotion in the perceiver (Carr et al., 2003; Rizzolatti and Craighero, 2004; Molnar-Szakacs and Overy, 2006)? Based on what is known about neural responses to rhythm (Nozaradan et al., 2011; Fujioka et al., 2012), we propose a simple, if somewhat speculative, interpretation. Activation in mirror regions reflects resonance of endogenous cortical rhythms to exogenous musical rhythms. Activation increases as tempo increases because, as this neural circuit entrains to the musical rhythm it tracks the tempo (i.e., frequency modulations) of the performance (Herrmann et al., 2013). The frequency modulations themselves would represent violations of temporal expectancy (Large and Jones, 1999). The expressive performance also led to emotion and reward related neural activations (when compared with a mechanical performance that precisely controlled for melody, harmony, and rhythm, see Chapin et al., 2010). We hypothesize that the frequency modulation of mirror regions led to these activations (Molnar-Szakacs and Overy, 2006; Chapin et al., 2010). Thus, perhaps music directly couples into affective circuitry by exploiting resonant modes of cortical function, thereby creating the basis for affective communication

### INTRINSIC DYNAMICS, MUSICAL QUALIA AND COGNITIVE DEVELOPMENT

The preceding discussion suggests that at least some aspects of affective responses to music are deeply rooted in the intrinsic physics of the brain and body. If this is true, then neurodynamic investigations may ultimately explain how musical rhythms couple into neural circuits and modulate affective responses. But, could the neurodynamic approach explain musical qualia more generally? Consider the fundamental qualitative difference between pitch and rhythm. A simple acoustic click, repeated at 5 ms intervals, generates a pitch percept at 200 Hz. Increase the interval to 500 ms and the percept is that of a series of discrete events, with a pulse rate of 2 Hz. From a dynamical systems point of view, it makes perfect sense that the neural mechanisms brought to bear on the two stimuli may be similar; the difference is merely one of timescale. Yet from a phenomenological point of view, the two are fundamentally different: a single continuous event versus a rhythmic sequence. Why the difference in qualia? Perhaps it is because the timescale at which distinct neural events are bound together into unified conscious scenes lie between these timescales of pitch and rhythm. Perhaps the difference in qualia lies in the timescale relationship, not in the mechanism* per se*. If so, perhaps neural oscillation explains not only rhythm related responses, but also pitch related responses, such as stability and attraction.

We have argued elsewhere that the terms stability and attraction, used by theorists to describe scale degree qualia (Meyer, 1956; Zuckerkandl, 1956; Lerdahl and Jackendoff, 1983; Lerdahl, 2001), are not metaphorical. These refer to real, dynamical stability and attraction relationships in a neural field stimulated by external frequencies (Large, 2010a; Large and Almonte, 2011). In other words, scale degree qualia are simply what it feels like when our brains resonate to tonal sequences. This approach can explain the perception of tonal stability and attraction in Western modes (Krumhansl and Kessler, 1982; Large, 2010a), and North Indian raga (Castellano et al., 1984; Large and Almonte, 2011). It may also shed light on the development of statistical regularities in tonal melodies, implying that certain pitches occur more frequently because they have greater dynamical stability in underlying neural networks.

There is now a great deal of evidence regarding development of basic music structure cognition, including meter (Hannon and Trehub, 2005; Kirschner and Tomasello, 2009; Winkler et al., 2009) and tonality (Trainor and Trehub, 1992; Schellenberg and Trehub, 1996; Trehub et al., 1999). Such results reveal developmental trajectories that occur over the first several years of life, as well as perceptual invariants that are consistent with intrinsic neurodynamics (Large, 2010b) tuned with Hebbian plasticity (Hoppensteadt and Izhikevich, 1996; Large, 2010a). Dalla Bella et al. (2001) asked if children can determine whether music is happy or sad. 3- to 4-year-olds failed to distinguish happy from sad above chance, 5-year-olds” responses were affected by tempo, while 6- to 8-year-old children used both tempo and mode. Thus, children begin to use tempo at about the same time the ability to synchronize movements emerges, and they begin to use mode at about the same time that sensitivity to key emerges (Trainor and Trehub, 1992; Schellenberg and Trehub, 1999; McAuley et al., 2006). The fact that the development of the two main musical dimensions – rhythm and tonality – have the same time course as their affective correlates, strongly suggests a link between the development of neurodynamic responses and music-induced affective experience.

We do not claim that musical qualia are hard-wired, however, our argument does suggest that substantive aspects of musical expectancy and musical contagion may be explainable directly in neurodynamic terms, linking “high-level” perception with “low-level” neurodynamics. In combination with Hebbian plasticity, intrinsic neurodynamic constraints could explain the sensitivity of infant listeners to musical invariants, as well as the ability to acquire sophisticated musical knowledge. Moreover, this explanation suggests that association of affective responses with the musical modes of diverse cultures may not be due entirely to convention, as has been speculated previously (Meyer, 1956; Huron, 2005). Indeed cross-cultural studies in the perception of Western music suggest that happiness and sadness are communicated, at least in part, based on mode (Balkwill et al., 2004; Fritz et al., 2009). Moreover, unencultured Western listeners may be able to understand the moods intended by Indian raga performances (Balkwill and Thompson, 1999; Chordia and Rae, 2008). At the very least, these cross-cultural findings suggest that associations of mood with mode have been prematurely dismissed as conventional, and these relationships deserve to be reevaluated.

### WHAT IS COMMUNICATED – BASIC EMOTION OR CORE AFFECT?

Juslin and Vastfjall (2008) propose that emotional contagion operates similarly to facial expression of basic emotions (Juslin and Laukka, 2003; Laird and Strout, 2007). However, because the theory of basic emotions has recently been called into question, it makes sense to review the body of evidence that pertains to music. In communication studies, both performances and listener judgments of intended emotion have been linked to specific musical features, including tempo, articulation, intensity, and timbre (Gabrielsson and Juslin, 1996; Peretz et al., 1998; Juslin, 2000; Juslin and Laukka, 2003). However, these studies also show that, at least for Western music, happiness and sadness are the most reliably communicated emotions (Kreutz et al., 2002; Lindström et al., 2002; Kallinen, 2005; Kreutz et al., 2008; Mohn et al., 2011), while other “basic” emotions are more often confused (Gabrielsson and Juslin, 1996; Peretz et al., 1998; Juslin, 2000; Juslin and Laukka, 2003; Kreutz et al., 2008; Mohn et al., 2011). Interestingly, Ekman (1993) and Izard et al. (2000) have both questioned the theory of facial expression of basic emotion based on variability and confusability. Moreover, analyses of physiological responses to music show that while musical stimuli elicit significant responses, physiological measures do not generally match listener self-reports using emotion terms (Krumhansl, 1997).

Basic emotion theory has been linked to an approach in which music is supposed to somehow imitate or mimic more biologically relevant stimuli, such as speech or mother-infant interactions (Juslin and Laukka, 2003), leading to the direct perception of emotion. Such discussions generally assume that musical communication is not evolutionarily selected, but needs to piggyback on more fundamental mechanisms. Our proposal is that music speaks to the brain in its own language, it need not imitate any other form of communication. In this sense, other forms of communication may be seen to induce or modulate emotions more indirectly, i.e., the effect is more cognitive (cf. Langer, 1951). Thus, the study of music may provide a unique window into the fundamental nature of affective communication, which might explain, for example, why music has the ability to evoke emotional memories (Janata et al., 2007).

It is tempting to try to unify core affect with basic emotion (Juslin, 2001) by assuming that each emotion category is associated with a specific core affective state (e.g., fear is unpleasant and highly arousing, sadness is unpleasant and less arousing, etc.). However, the mapping of emotion to affect is not unique; core affective states experienced during two different episodes of a given, nameable emotion (e.g., fear) typically differ depending on the situation (Meyer, 1956; Barrett, 2009b; Wilson-Mendenhall et al., 2013). Moreover, musical variables such as melodic contour, tempo, loudness, texture, and timbral sharpness, predict real-time listener ratings of arousal and valence well (Schubert, 1999, 2001, 2004), and correlate with BOLD responses in a number of brain regions (Chapin et al., 2010). Neuroimaging studies have also revealed BOLD responses to parametric manipulation of pleasantness (Blood et al., 1999; Koelsch et al., 2006), and these overlap with responses to intensely pleasurable music (Blood and Zatorre, 2001; Salimpoor et al., 2010).

## CONCLUSION

In summary, we believe that a coherent picture is developing, based on recent findings of nonlinear resonant responses to acoustic stimulation at multiple timescales (Ruggero, 1992; Joris et al., 2004; Lakatos et al., 2005; Lee et al., 2009; Laudanski et al., 2010; Nozaradan et al., 2011) and theoretical analyses that show how such processes could underlie complex cognitive computations as well as phenomenal and affective aspects of our musical experiences (Baldi and Meir, 1990; Hoppensteadt and Izhikevich, 1997; Izhikevich, 2002; Shapira Lots and Stone, 2008; Large, 2010b). Such results and analyses suggest that neurodynamics provides an appropriate level at which to understand not only perceptual and cognitive responses to music, but ultimately affective and emotional responses as well. We suggest that, to support affective communication, music need not mimic some other type of social interaction; it need only engage the nervous system at the appropriate timescales. Indeed, music may be a unique type of stimulus that engages the brain in ways that no other stimulus can.

Thus, we suggest that there is something special about the way music communicates emotion. Our approach recasts musical expectancy and affective contagion as nonlinear resonance to musical patterns. Resonance occurs simultaneously on multiple timescales, leading to stable or metastable patterns of neural responses. Such patterns are inherently spatiotemporal, however, temporal aspects of the stimulus determine at any specific point which neural structures are involved. Violations of expectancy, such as the occurrence of a strong rhythmic event on a weak beat (a syncopation) or the prolongation of an unstable tone where a stable tone is expected (an appoggiatura), would correspond to a disruptions, or perturbations of the ongoing pattern. Implication and realization would correspond to relaxation toward, and reestablishment of a stable orbit. Stable and unstable in this context, are determined by the intrinsic neurodynamics of brain networks involved, which depend, in part, on tuning of the dynamics via synaptic plasticity. In this way, music may modulate affective neurodynamics directly by coupling into those aspects of the dynamic core of consciousness that govern our subjective feelings from moment to moment.

## GLOSSARY

**Affect/Core affect:** The most elementary consciously accessible sensation evident in moods and emotions*. *Affect can be characterized as fluctuating level of valence (pleasure/displeasure) and arousal (activation/ deactivation; Barrett and Bliss-Moreau, 2009). Core affect is so called because it is thought to arise in the core of the body or in neural representations of body state change (Russell, 2003).

**Basic emotions:** A few privileged emotion kinds (e.g., anger, sadness, fear, and happiness), each of which is thought to derive from an evolutionarily preserved brain module. Basic emotions are discrete, and each category shares a distinctive collection of properties, including patterns of autonomic nervous system activity, behavioral responses, and action tendencies. A set of emotion-specific brain structures is thought to mediate these particular “basic” emotions (Allport, 1924; Izard, 1971; Ekman, 1972; Panksepp, 1998).

**Dynamic core:** Functional clusters of neuronal groups in the thalamocortical system that are hypothesized to underlie consciousness. Distinct neuronal groups contribute to the contents of consciousness through enhanced synchrony of neural rhythms. The boundaries of this core are suggested to shift over time, with transitions occurring under the influence of internal and external stimulation (Seth, 2007).

**Empathy: **A feeling that arises when the perception of an emotional gesture in another person directly induces the same emotion in the perceiver without any appraisal process (see Juslin and Vastfjall, 2008).

**Emotional contagion/Affective contagion: **A process that occurs between individuals in which emotional or affective information is transferred from one individual to another. The idea that people may “catch” the emotions of others when seeing their facial expressions, hearing their vocal expressions, or hearing their musical performances (see Juslin and Vastfjall, 2008)

**Emotion:** Affective responses to situations that usually involve a number of sub-components – subjective feeling, physiological arousal, thought, expression, action tendency, and regulation – which are more or less synchronized (Juslin and Vastfjall, 2008). Emotions are intentional; they are about an object or event.

**Feelings:** The subjective phenomenal character of an experience, used informally to refer to qualia or affect.

**Intentionality:** The power of minds to be about, to represent, or to stand for, things, properties and states of affairs (Jacob, 2010).

**Psychological constructionism: **The theory that emotions results from the combination of psychologically primitive processes, which encompass both affective and intentional components. A specific emotion is not the invariable result of activation in a particular brain area; neural circuitry realizes more basic processes across emotion categories. Psychological constructionists argue that emotions are culturally relative and learned (see Russell and Barrett, 1999; Barrett and Bliss-Moreau, 2009; Barrett, 2009a,b).

**Qualia/musical qualia:** The distinctive subjective character of a mental state; what it is *like* to experience each state; the introspectively accessible, phenomenal aspects of our mental lives (Tye, 2013). Musical qualia refers to the subjective character of specific musical events, experienced within a tonal and/or temporal context.

## Conflict of Interest Statement

The authors declare that the research was conducted in the absence of any commercial or financial relationships that could be construed as a potential conflict of interest.

## Footnotes

1 We use the word intentionality to refer to any mental phenomena that have referential content (see glossary).

2 f1 and f2 denote frequencies of pure tones and k and m are positive integers.
